# Warm summers during the Younger Dryas cold reversal

**DOI:** 10.1038/s41467-018-04071-5

**Published:** 2018-04-24

**Authors:** Frederik Schenk, Minna Väliranta, Francesco Muschitiello, Lev Tarasov, Maija Heikkilä, Svante Björck, Jenny Brandefelt, Arne V. Johansson, Jens-Ove Näslund, Barbara Wohlfarth

**Affiliations:** 1 Bolin Centre for Climate Research and Department of Geological Sciences, Stockholm University, Svante Arrhenius väg 8, SE106-91 Stockholm, Sweden; 2 Department of Mechanics, Linné FLOW Centre, KTH Royal Institute of Technology, Osquars backe 18, SE100-44 Stockholm, Sweden; 3 Environmental Change Research Unit (ECRU), Ecosystems and Environment Research Programme, Faculty of Biological and Environmental Sciences and Helsinki Institute of Sustainability Science (HELSUS)​, University of Helsinki, P.O. Box 65, 00014 Helsinki, Finland; 4 Department of Geography, University of Cambridge, Cambridge, CB2 3EN UK; 5 Lamont-Doherty Earth Observatory, Columbia University, 61 Route 9 W, Palisades, New York, NY 10964-8000 USA; 6 Department of Physics and Physical Oceanography, Memorial University of Newfoundland, St. John’s, NL A1B 3X7 Canada; 7 Department of Geology, Quaternary Sciences, Lund University, Box 117, SE221-00 Lund, Sweden; 8 Swedish Nuclear Fuel and Waste Management Company (SKB), Box 250, SE101-24 Stockholm, Sweden; 9 Department of Physical Geography and Quaternary Geology, Stockholm University, Svante Arrhenius väg 8, SE106-91 Stockholm, Sweden

## Abstract

The Younger Dryas (YD) cold reversal interrupts the warming climate of the deglaciation with global climatic impacts. The sudden cooling is typically linked to an abrupt slowdown of the Atlantic Meridional Overturning Circulation (AMOC) in response to meltwater discharges from ice sheets. However, inconsistencies regarding the YD-response of European summer temperatures have cast doubt whether the concept provides a sufficient explanation. Here we present results from a high-resolution global climate simulation together with a new July temperature compilation based on plant indicator species and show that European summers remain warm during the YD. Our climate simulation provides robust physical evidence that atmospheric blocking of cold westerly winds over Fennoscandia is a key mechanism counteracting the cooling impact of an AMOC-slowdown during summer. Despite the persistence of short warm summers, the YD is dominated by a shift to a continental climate with extreme winter to spring cooling and short growing seasons.

## Abstract (web summary)

Mechanisms causing the Younger Dryas cold reversal have been questioned by inconsistencies between proxy and modelling results. Here, the authors show that the concept of a strong North Atlantic Ocean cooling event as major driver is consistent with warm European summers caused by intensified atmospheric blocking.

## Introduction

Proxy records from the Euro-Atlantic region indicate that the Younger Dryas (YD) cold reversal lasted from ~12.8 to 11.68 thousand years ago (ka). The period is dominated by a cooling of the North Atlantic Ocean and expansion of sea ice^1–5^, marked changes in Northern Hemisphere atmospheric circulation^4,6^ with a shift to regionally dry conditions^1,7–9^ and an overall more continental climate. Although the strongest cooling can be attributed to the winter season^6^, fossil assemblages of chironomid larvae (non-biting midges)^10,11^ also indicate several degrees colder European summer temperatures despite the very high and continuously increasing summer insolation over northern latitudes^12^. Some studies suggest however that summer conditions during the YD might not have been particularly cold at least locally^13–18^.

The ca. 1100-year-long cold period is typically explained by an abrupt shift to a weaker state of the Atlantic Meridional Overturning Circulation (AMOC)^19–22^ in combination with atmosphere–sea-ice–ocean feedbacks^8,9^. The reason for the abrupt slowdown of the AMOC has been attributed to the excess freshwater fluxes into the North Atlantic Ocean in response to melting ice sheets^9,21,22^. Coupled atmosphere–ocean models, simulating a strong AMOC reduction during the YD^11,21,23–26^, generally reproduce the winter-dominated annual cooling signal in Greenland ice cores^4,27^ but fail to reproduce the European summer cooling^11,28^. It was hence suggested that forcings other than the AMOC slowdown and the associated cooling of the North Atlantic Ocean are required to explain the YD such as a reduction in solar activity^28^, a change in wind patterns^29^ or ice sheets^30,31^, or possibly a combination of an AMOC slowdown with lower solar activity^11^. As evidence for such a ~1100-long negative forcing is lacking, the mechanisms and/or regional climatic impacts of the YD remain elusive.

The current lack of understanding and consistency regarding the YD cold reversal raises several questions. From a climate modelling perspective, a key question is whether the assumed atmospheric and oceanic forcings and/or model boundary conditions^11,30^ are realistic. In this context, it is also unclear to which extent current coarse resolution climate simulations (e.g. refs. ^25,26,32–34^) can realistically reproduce the regionally complex YD climate response depicted in proxy data^35,36^. From a climate proxy-perspective, the question remains whether other factors than summer temperature per se^37–40^ are responsible for the cold summer temperatures reflected in most European fossil chironomid assemblages^10,11^ or, e.g. the widespread regional decline or disappearance of trees^18^.

To further investigate the regional YD climate response relative to the preceding warm Bølling-Allerød (BA) period, we present here high-resolution climate simulations of the BA interstadial and YD stadial. In contrast to previous coarse resolution modelling studies^9,25,26,32–34^, this allows us to study in unprecedented detail how atmospheric circulation and related summer temperatures react to the very strong oceanic cooling in the presence of high orbital summer insolation. Consistent with new proxy evidence where we reconstruct July temperatures based on the climate indicator-plant species approach^41^, we find clear evidence for warm European summers during the YD as a result of persistent atmospheric blocking over the NE-Atlantic and Fennoscandian Ice Sheet.

## Results

### Paleo-ocean states and late-glacial ice sheets

**Fig. 1:**
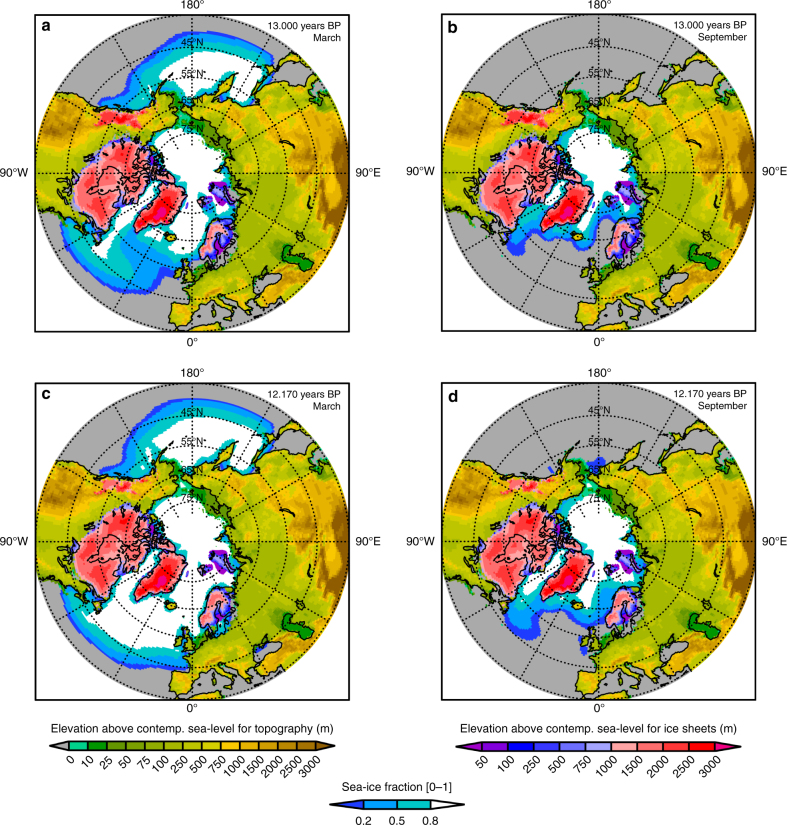
Late-glacial topography, ice sheets and sea-ice extent for BA and YD. The sea-ice extent represents here the simulated winter maximum (**a**, **c**, March) vs. summer minimum (**b**, **d**, September) for the late BA interstadial (**a**, **b**) and mid-YD stadial (**c**, **d**). The simulated change in sea-ice extent corresponds to an AMOC reduction from BA to YD of around −36% based on CCSM3^25,26^. The paleotopography is consistent with contemporary sea-level stands and updated ice sheet reconstructions of GLAC-1B (^42^, Methods)

For our high-resolution (0.9° × 1.25°) global climate simulations for the BA and YD, we set up an interactively coupled atmosphere–land-ice-model configuration with prescribed ocean conditions using the Community Earth System Model (CESM 1.0.5). Here, we focus on two time slices: the final part of the warm BA period (13 ka) and the mid-YD cold state (12.17 ka). The simulations are performed using modified realistic boundary and forcing conditions (Methods) taking into account a new reconstruction of continental ice sheets (GLAC-1B)^42^(Methods, Fig. 1), a land-sea distribution with a 60–70 m lower sea-level, and orbital and greenhouse gas forcing for BA and YD, respectively (Supplementary Table 1). The model is forced by the strong oceanic cooling and expansion of sea-ice during the YD reflecting global sea-surface temperatures (SSTs) in response to a slowdown of the AMOC of ~36% (−5.27 Sv) during the YD compared to the preceding BA^25,26^, consistent with geological evidence^22^. The large increase in simulated maximum (March) and minimum (September) sea-ice extent during the YD (Fig. 1c, d) constitutes the dominant forcing for our high-resolution atmosphere–land–sea-ice simulation. Relative to the BA, the strong mid-YD oceanic cooling signal over the North Atlantic competes with a 5 W/m^2^ (+1.06%) increase in summer radiation due to orbital forcing at 60° N[12], with only a minor negative change in radiation of −0.18 W/m^2^ or −7.75 CO_2_-equivalent due to Greenhouse gas (GHG) forcing (Supplementary Table 1).

### Multi-proxy July temperature reconstructions

In addition to our climate simulations, we have compiled a comprehensive multi-proxy data set of quantitative European summer temperature reconstructions based on 122 lake sediment records (Supplementary Fig. 1; Supplementary Data 1), which cover the periods of BA and YD. Here we introduce an approach with climate indicator-plant species as an independent July temperature proxy^41^. This enables testing the consistency of cold YD summer conditions predicted by fossil chironomid assemblages used in previous studies^10,11^.

The new compilation of climate indicator-plant species is based on published macrofossil and local pollen records (Supplementary Fig. 1; Supplementary Data 1) covering most of Europe. Here, we only include plant species that indicate local presence focusing primarily on specific plant species, such as aquatics and riparian species (Supplementary Table 2). These species show rapid and clear temperature responses (Methods) and are not overly sensitive to changes in other environmental drivers once the population is established^43^. In the following, we use the multi-proxy-based July temperature differences (Δ*T*) of YD minus BA for comparison with the simulated summer temperature response by CESM1.

### Persistent warm summers during the Younger Dryas

**Fig. 2:**
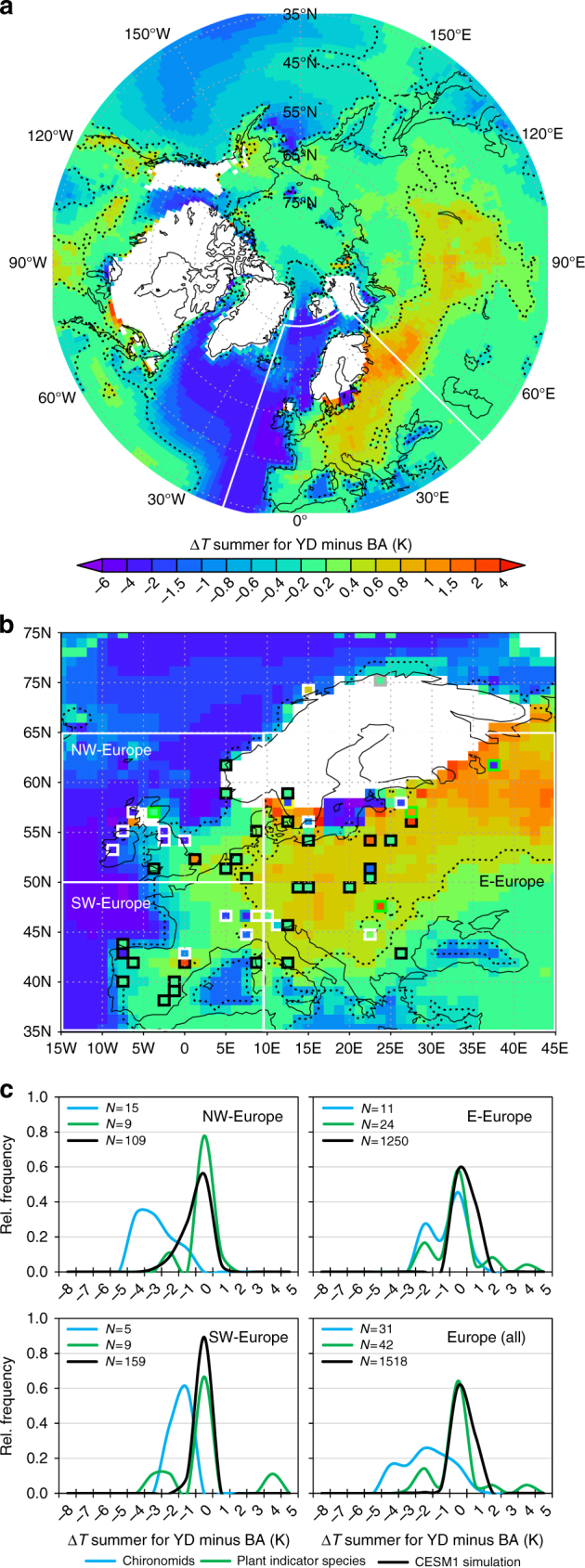
Summer temperature differences for YD minus BA. **a** Simulated summer (JJA) temperature differences (K) over the Northern Hemisphere and **b** over Europe in comparison to multi-proxy July temperature differences based on chironomids (white rectangles), aquatic (black rectangles) and terrestrial (green rectangles) climate indicator-plant species. **c** Relative spatial frequencies of temperature anomalies (K) across different European regions derived from chironomids (blue), plant indicator species (green) and the CESM1 model simulation (black). Dashed lines in **a** and **b** indicate areas with significant temperature deviations (*p* < 0.05). See Supplementary Fig. 1 for a more detailed proxy-type comparison and proxy-based mean temperatures

Our model simulation shows that the response of terrestrial summer temperatures to the competing effects of extended sea-ice (Fig. 1c, d), with a 4–6 K colder North Atlantic (Fig. 2a, b) and increased orbital summer forcing, does not lead to cooling over continental Europe (Fig. 2). Our high-resolution CESM1 simulation hence confirms the absence of a YD summer cooling predicted by previous coarse resolution simulations^11,28^ with the exception of CCSM3^25,26^. CESM1 shows a significant YD summer warming of 0.4 to 1.5 K compared to BA over central to eastern continental Europe. Cooling only occurs in near-coastal areas of Western Europe (Fig. 2b).

The spatial comparison of simulated temperatures with the multi-proxy-based summer temperature anomalies for YD minus BA yields a heterogeneous picture (Fig. 2b). The spatial frequency distributions for the occurrence of different local temperature changes (Δ*T*) within different regions derived from our plant proxies shows that July temperature anomalies compare well to the simulated summer temperature response, in contrast to the fossil chironomid estimates (Fig. 2c). The latter display a clear meridional gradient^10^ with an up to 4.3 K cooling over NW-Europe (median of regional Δ*T* = −3 K), but less over continental E-Europe (median Δ*T* = −0.3 K) compared to BA (Fig. 2c). In agreement with our simulation, our plant indicator species do not show such a cooling gradient and imply little to no temperature changes at most sites (median Δ*T* = 0 K for all regions) (Fig. 2c). Relatively warm YD summer temperatures and hence little to no changes relative to BA, as reconstructed from indicator plants, are well within the spread of our simulated summer (JJA) temperature response (Fig. 2c) and suggest that these reflect ambient air temperatures.

The discrepancy of up to several degrees cooling over NW-Europe (chironomids) compared to little or no cooling (plant indicators and CESM1) (Fig. 2c) clearly exceeds the typical uncertainties of chironomid-based July temperatures of around 1.4 K^10,11^ (Methods). Although the indicator approach does not provide comprehensive statistical error estimates^41^, several factors suggest that our temperature reconstruction based on plant indicator species will rather underestimate July temperatures during the YD (see Methods).

The consistency of the plant species data, which represents various habitats, with our model simulations lends credibility to their ability to replicate late-glacial conditions. The persistence of warm July temperatures are further supported as our plant indicator species-based reconstructions were mainly derived from the presence of seeds of particular species, which suggest ongoing sexual reproduction and thriving populations through the YD. Thus, the data does not reflect individuals persisting in vegetative, non-reproducing stage^46,47^. We therefore underline that all plant species used here primarily reflect temperature^41^ (Methods) with a low sensitivity to other environmental changes once the population is established^43^. It should be noted however that the present-day distribution pattern of many plant species included in our database shows that the minimum July temperature requirement varies depending on continentality. For example, the northern distribution limit of *Typha latifolia* in Fennoscandia follows the 15.7 °C July temperature isoline while it corresponds to the 17 °C July temperature isoline on the Russian Plain^41^. Thus, our temperature reconstructions derived from plant species distribution–climate relationships in Finland may actually yield in underestimations, i.e. too cold July temperature reconstructions for the more continental climate of the YD. This would explain why the plant indicator species do not capture the slight simulated warming of ~0.5 to 1.5 K over the very continental regions of E-Europe during the YD (Fig. 2c).

### The dominance of seasonality changes

But how can the strong YD summer cooling signal be explained which seems to dominate reconstructions based on chironomid assemblages (Fig. 2c; Supplementary Fig. 1)? An important factor might be, in contrast to the used plant species, that chironomid assemblages have been shown to incorporate a number of environmental signals (e.g., catchment vegetation, nutrient supply, lake status and depth, seasonality) apart from the ambient summer air temperature^37–40^. A major factor related to proposed YD changes might be that significant changes in seasonality will have profound impacts on the duration of their different life cycle stages. We therefore hypothesise that other environmental factors than summer temperatures alone played a vital role for the chironomid assemblages at the BA-YD transition. To address this hypothesis, we test here to which extent simulated changes in seasonality between a warm (BA) and cold (YD) climate state can explain the cooling pattern depicted by chironomids.

**Fig. 3:**
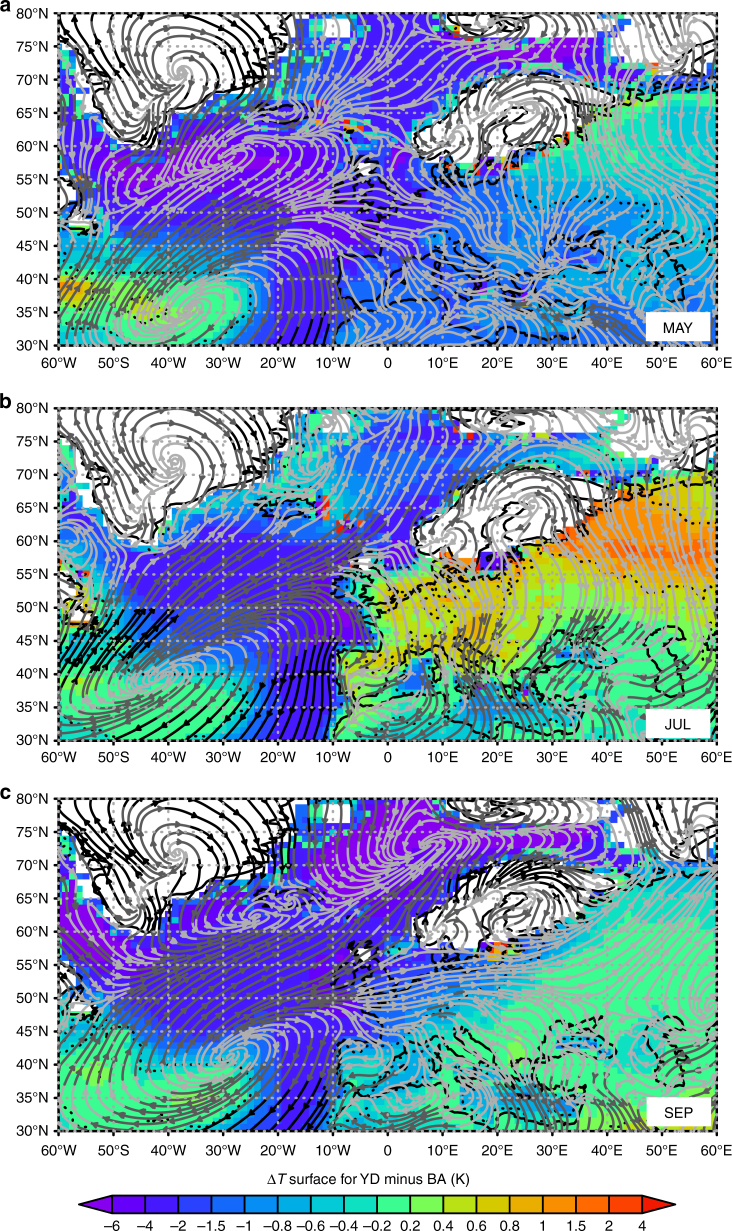
Simulated link between YD-BA temperature changes and mean atmospheric flow during the YD. Simulated changes in monthly mean surface temperatures of YD minus BA (K) with CESM1 for **a** May, **b** July and **c** September (shaded colours). May and September show a strong cooling from W to E-Europe which is in contrast to the warm but very short summer season. Stream lines represent the monthly mean wind patterns during the YD which highlight the cooling by zonal advection in May and September from the cold North Atlantic towards Europe which is absent during summer

**Fig. 4:**
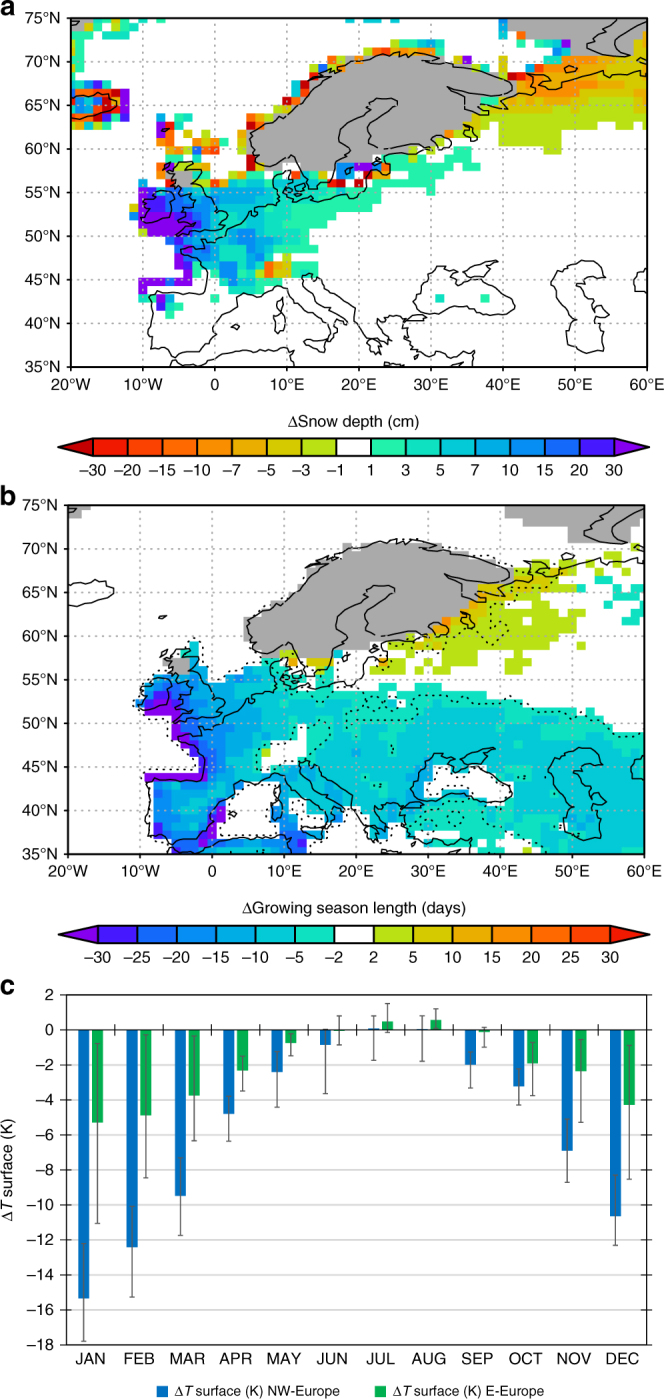
Simulated seasonality changes during the YD relative to BA. **a** Changes in snow depth (cm) in May; **b** changes in GSL (days) and **c** changes in the annual cycle of monthly mean surface temperatures averaged over NW-Europe (blue) and E-Europe (green) (K). Error bars in **c** represent the 90% spatial temperature spread around the regional mean temperature change

Indeed, warm summers in our simulation are restricted to July and August only (Figs. 3 and 4c), whereas strong cooling lasts until late May or early June (Fig. 3a). Significant cooling starts again in September (Fig. 3c) with severe cooling during the winter season (Fig. 4c). As shown by the stream lines representing the mean monthly wind patterns during the YD in Fig. 3a–c, the cooling pattern in May and September shows a strong link to dominating westerly winds advecting much colder air from the North Atlantic compared to the warmer BA ocean state. With exception of the summer months (JJA, Figs. 2b and 4c), the coupling between cold temperatures with westerly winds allows only a very short warm-summer episode across Europe (Fig. 4c) with overall strong cooling before and after (Fig. 3).

As a result, the higher snow depths over NW-Europe which last until May (Fig. 4a) and the significant decrease in growing season length (GSL, N days per year ≥ 5 °C) by up to several weeks over western to central Europe (Fig. 4b) imply extreme potentially non-analogue changes in seasonality with strong impacts on most terrestrial and aquatic ecosystems. The spatial pattern with a meridional gradient of strong cooling in the NW and less so towards the E as shown by chironomid-based temperature estimates (Fig. 2c; Supplementary Fig. 1c) compares well to the simulated cooling pattern in May (Fig. 3a) with increased snow depth, i.e. a shortening of the warm period and shorter GSL (Fig. 4).

Additional differences between the two proxy-based temperature data sets appear when elevation-corrected regionally averaged median July temperatures during BA and YD are compared to each other. Plant-based median July temperatures appear systematically higher than those derived from chironomid assemblages in both periods (Supplementary Fig. 2). However, while chironomid-based median BA July temperatures are only 1.2 K to 1.6 K colder than plant-based temperatures over NW-Europe, the difference strongly increases during the YD with chironomids being on average more than 4 K colder than plant-based estimates (Supplementary Fig. 2). This suggests that the pronounced changes in seasonality and GSL (Fig. 4) during a colder climate state have significant impacts on chironomid assemblages but not on the climate indicator-plant species used in this study.

This leads to the conclusion that chironomid-based temperatures rather reflect the simulated shift in seasonality to cold late spring temperatures (Fig. 3a) and/or shorter warm season (Fig. 4) while the persistence of warm summers (Figs. 2 and 3b) during the YD is consistent with climate indicator-plant species. Qualitative uncertainty estimates for the climate indicator-plant species (Methods) suggest that summers during the more continental YD might have been even warmer. This would hence further increase the mismatch between chironomids and plant indicator species during the YD but would bring plant indicator species-based temperatures closer to the CESM1 simulation. The results of the comparison made here clearly motivate further multi-proxy approaches to better understand the potential impact of extreme seasonality changes on different (a)biotic proxies.

### Atmospheric blocking during summer

The consistent finding of relatively warm summers during the YD cold reversal, based on our plant indicator species approach and our high-resolution simulation, has profound consequences for understanding the driving forces and physical-dynamical response of the climate system to a cold-ocean state like the YD. On the basis of our climate simulations, we can identify four mechanisms which all favour the persistence of warm summers during the YD.

**Fig. 5:**
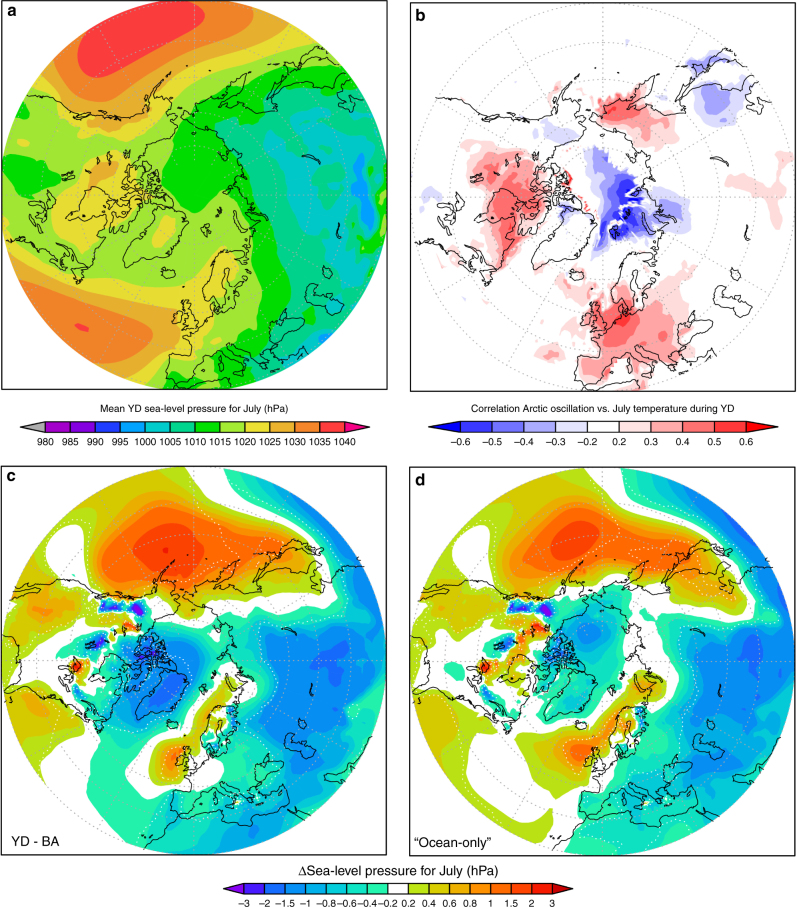
Simulated atmospheric blocking and Arctic teleconnection. **a** Mean SLP pattern during the YD with a ridge extending from the Azores over the Fennoscandian Ice Sheet. **b** Correlation of mean July surface temperatures with the Arctic Oscillation (AO) (EOF1 of SLP north of 25°N). **c** Changes in monthly mean SLP of YD minus BA showing a strengthening of the pressure ridge over the E-Atlantic. **d** Same as **c** but without changes in radiation (“cold-ocean only effect”). Lower pressure over the Arctic during YD (**c**) resembles a positive AO which is positively correlated with warming temperatures over Europe (**b**)

First, the most dominant mechanism to support warm-summer conditions refers to the mean sea-level pressure (SLP) state during summer. The mean sea-level pressure pattern is characterised by a high-pressure ridge, connecting the Azores High with the Fennoscandian Ice Sheet (FIS) (Fig. 5a). During summer (JJA), this leads to atmospheric blocking, preventing cool westerly winds from the North Atlantic from entering continental Europe (Fig. 3b).

Although this phenomenon also exists in the BA simulation—and most likely during the whole deglaciation—the high-pressure ridge and hence atmospheric blocking is further strengthened over the NE-Atlantic (Fig. 5c) in response to the strong ocean cooling during the YD. A second regional factor for the persistence of warm YD summers is hence related to a thermodynamically induced stronger atmospheric blocking of cold westerly winds (Fig. 3b). The counterintuitive climatic response, that very cold North Atlantic SST’s like during the YD induce warmer summers over central to E-Europe, has recently also been identified as an important driver behind major European heat waves since the 1980s which occurred during unusually cold SST’s over the North Atlantic^44^. The recent European drought of 2015 may provide a modern ‘analogue’ of the YD where relatively dry conditions with low soil moisture start a positive feedback for intensified warming in late summer over central to E-Europe associated with persistent atmospheric blocking events supported by strong negative SST anomalies over the central North Atlantic. The observed cold-ocean–warm-summer response is consistent with our YD simulation (compare also Supplementary Fig. 5) and suggests that such a thermodynamic response of the atmosphere in combination with relatively dry conditions such as during the YD^1,7–9^ is physically consistent with warmer summers as captured by our European plant indicator species and climate simulation. More detailed studies are however needed to further explore the importance and regional relevance of cold North Atlantic SSTs on European summer temperatures both, in the modern climate as well as for different climate periods in the past.

A third mechanism supporting warm-summer conditions during the YD relates to a shift in the teleconnection pattern known as the Arctic Oscillation (AO). The AO, which can be defined as the first leading principal component (PC1) of the pressure field variability north of 20°N, links changes in the zonal pressure gradient relative to the Arctic with variations in northern hemispheric climate. For Europe, the dipole of SLP changes during the YD with lower SLP over the Greenland–Arctic region and higher SLP over the NE-Atlantic (Fig. 5c) implies a shift to a stronger zonal pressure gradient and hence a positive mode of the AO. As shown in Fig. 5b, a positive AO is strongly correlated with positive summer temperature anomalies over Europe. The shift to AO+ in response to cold summer SSTs is hence another large-scale factor for warmer summers during the YD.

It should be noted here that the Arctic–European dipole of SLP changes during the YD (Fig. 5c) is a robust thermodynamic response to the ocean cooling which is also simulated by a coarse resolution climate model^9,28^. However, the coarse resolution typically hinders these models to realistically simulate the related atmospheric circulation. Consequently, the transient but coarse resolution simulation of the YD with CCSM3^9,25,26^, which is identical to our simulation with respect to the horizontal boundary conditions of the prescribed ocean state and radiative forcing, does not, in spite of a comparable pressure-dipole^9^, capture the atmospheric blocking and deflection of westerly flow by FIS during summer (Supplementary Fig. 3). As a result, the coarse resolution model of CCSM3 simulates westerly flow with a direct advection of cold air from the North Atlantic also during summer with the result of substantial cooling during the YD (Supplementary Fig. 4). The warm summers predicted by our climate indicator-plant species in this study hence provide a plausible confirmation that atmospheric blocking, as simulated here with CESM1, was indeed a dominant factor also during the YD cold reversal.

A forth factor contributing to warm-summer conditions during the YD relates to the high and continuously increasing orbital summer insolation over high northern latitudes^12^. A sensitivity experiment, where we repeat the simulation of the mid-YD cold-ocean state, but without any increase in radiative forcing relative to the BA, indicates that a very cold North Atlantic Ocean alone causes already slightly warmer central European summers (Supplementary Fig. 5). This cold-ocean–warm-summer mechanism can be explained by strengthened atmospheric blocking over the NE-Atlantic (Fig. 5d) in line with observations related to recent heat waves and droughts during anomalous cold SSTs^44,45^. The increasing summer insolation alone (residuals in Supplementary Fig. 5) contributes mainly to the warming over continental Eurasia while the effect of intensified atmospheric blocking dominates mainly regionally over central Europe.

## Discussion

On the basis of our high-resolution simulations of the late-glacial period with CESM1, we find clear evidence that persistent atmospheric/orographic blocking induced by the Fennoscandian Ice Sheet (FIS) plays a key role for the European summer climate. In addition to such a persistent blocking of cold westerly flow by FIS during BA and YD, we can identify three additional factors captured by our high-resolution simulation, ranging from local thermodynamic effects in response to strong oceanic cooling to large-scale atmospheric teleconnections and increased summer insolation. These factors provide a physically robust explanation for the persistence of warm-summer conditions also during the YD cold reversal. Our simulations confirm previous coarse resolution model simulations which, even without atmospheric blocking by FIS, suggested warm summers during the YD despite a strong AMOC-slowdown and related North Atlantic cooling^11,32,34^.

On the basis of the consistency of our model simulation with summer temperature estimates from our climate indicator-plant species, we argue that the simulated atmospheric blocking of cold westerly winds provides a key mechanism to reproduce late-glacial summer climates including the YD cold state. The plant macrofossil results in our compilation shows continued sexual reproduction of the set of climate indicator-plant species, confirming that summers during the YD remained at least as warm as during the preceding BA warm period.

The spatially extended proxy- and model-evidence presented in this study is consistent with multiple previous local to regional studies which predicted warm-summer conditions during the YD stadial^13–18^. The multi-proxy confirmation of European-scale persistence of warm summers during the YD suggests that high orbital forcing during summer^12^ in combination with atmospheric blocking dominate also stadial conditions during the late glacial. The additional strengthening of atmospheric blocking in response to cold North Atlantic SSTs, as simulated by CESM1 during the YD (Fig. 5c, d; Supplementary Fig. 5), has found support by observations linking recent European heat waves and droughts to unusually cold North Atlantic SSTs^44,45^. Although more research is required to better understand the cold-ocean–warm-summer link on different timescales, this mechanism suggests that also the proxy evidence for regionally dry conditions during the YD^1,7–9^ might be an additional key factor which impacted the climate and, i.e. terrestrial ecosystems during stadial conditions.

Results from our study together with previous proxy-^13–18^ and modelling studies^11,32,34^ do not support the interpretation that the cooling signal found in various other European proxies like chironomids^9–11^ or the widespread disappearance of European forests^18^ reflect peak summer conditions during the YD. However, this cooling signal is indicative of another important aspect which dominates the stadial conditions of the YD. As shown by our CESM1 simulation, the magnitude and spatial pattern of cold bias reflected by chironomid assemblages relative to plant indicator species and our climate model results is consistent with the simulated cooling in late spring when atmospheric blocking is absent. The multi-proxy-model comparison therefore confirms previous interpretations that the strong change in seasonality, and hence a shift to more continental climate conditions^3,6^ dominates the climatic conditions during the YD.

In summary, our spatially detailed proxy-model comparison provides clear evidence that the YD cold reversal is characterised by short warm summers with comparably high summer temperatures as the preceding BA. The results hence support the hypothesis that a shift to extreme continentality^6^, with severe winters vs. warm short summers, explains the extreme climate during the YD. Our high-resolution climate simulation demonstrates that atmospheric blocking is a key mechanism to explain warm summers during the late glacial including the YD cold reversal. Short warm seasons in combination with severe winter–spring cooling and a fundamentally different seasonal cycle are hence consistent with a scenario where a strong AMOC-slowdown and related feedbacks are the dominant drivers.

## Methods

### Climate model CESM1.0.5

For our high-resolution global climate simulations of the pre-YD warm period (BA) and Younger Dryas (YD), we use version 1.0.5 of the Community Earth System Model (CESM1.0.5). The CESM1 versions are built on and hence include the last CCSM4.0 model version (CCSM4 = CESM1). Using a modular design, the full version (B component set) of CESM1.0.5 consists of interactively coupled models for the atmosphere (CAM), ocean (POP), land (CLM) and sea-ice (CICE), and is maintained by the National Center for Atmospheric Research (NCAR) in Boulder, Colorado, USA. The model code and a detailed documentation of CESM1 and its model components are available at http://www.cesm.ucar.edu/models/cesm1.0/.

The many different combinations of model components, grid versions and grid resolutions of CESM1 have been extensively validated against climate observations and are released as “scientifically supported model configurations”. In this study, we use a coupled atmosphere–land–sea-ice version (F component set) replacing the active ocean model with prescribed surface ocean conditions from a previous transient atmosphere–ocean simulation of CCSM3^25,26^. Our horizontal model resolution is 0.9° × 1.25° (~100 km) with a finite volume grid of 26 vertical levels for CAM and 15 vertical levels for the soil module in CLM.

CESM1 and the previous model version CCSM3 have been extensively used for simulations of the recent and deep past and have contributed to the modelling of the Last Glacial Maximum (LGM) and mid-Holocene climates in the PMIP and CMIP projects^48–51^. The multi-model evaluation against geological data from the past in PMIP2 demonstrated in general a good performance of these models regarding trends and large-scale patterns of climate variations in the past. On a regional scale, however, they tend to underestimate the magnitude of past changes as shown by comparisons to available paleo-data^51^. Further, simulations of other past periods that are fundamentally different from today’s climate have been performed with CCSM3 (e.g. Greenland Stadial 12 ~44 thousand years (kyr) ago^52^) and with CCSM4 (e.g. the Penultimate Glacial Maximum ~140 kyr ago)^53^. A transient atmosphere–ocean simulation with CCSM3 for the past 21 kyr has shown that the model captures large-scale climatic changes in good agreement with geological evidence^25,26,33^, including, for example, the bipolar seesaw with cooling over the Northern Hemisphere and warming in the Southern Ocean around Antarctica in response to variations in the Atlantic Meridional Overturning Circulation (AMOC)^54^.

### Ice sheet reconstruction and paleotopography

For simulations of the last deglaciation, fundamental changes to the horizontal boundary conditions of all model components are required to account for the presence of continental ice sheets, glacio-isostatic vertical land movements and changes in coastlines due to sea-level low stands. For our climate simulation, we make use of an interim (GLAC-1B) version of the GLAC1 ice sheet reconstruction. This version is a precursor of GLAC-1D^55^ that will be used for PMIP4 simulations and includes a different Eurasian component. Three of the component ice sheets have been published: North America (run nn9894)^42^, Antarctica (nn4041)^56^ and Greenland (Grb)^57^. The Eurasian component (nn36709) is from an ongoing Bayesian calibration of the 3D Glacial Systems Model (GSM) run at 0.5° latitude and 0.25° longitude. It uses the DATED^58^ Fennoscandian ice sheet retreat chronology, which is based on ice marginal features and comprehensive age determinations, and a climate forcing that incorporates results from the PMIP II and III model inter-comparison projects for the LGM. Like all components of GLAC, the deglacial nn36709 reconstruction is from a last glacial cycle (or longer) transient run. The GSM configuration for nn36709 is similar to that described in ref. ^42^, including a thermo-mechanically coupled glaciological ice sheet model (shallow ice approximation), visco-elastic isostatic response using the VM5a^59^ spherically symmetric earth rheology, bed thermal model (though in this case permafrost-resolving)^60^, asynchronously coupled surface drainage solver and surface and calving mass-balance modules. Aside from the ice sheet margin chronology, the main constraints for the calibration include relative sea-level records and present-day observed vertical velocities of the solid earth.

The land mask and sea level are a near gravitationally self-consistent solution of the sea-level equation that does not take into account rotational corrections but does account for pro-glacial lakes. Rotational corrections are <7 m at the LGM^61^ and therefore negligible near the ice-sheets given all the uncertainties in the reconstruction. The reconstruction is not able to reconcile near and far-field constraints for LGM ice volume and associated relative sea-level, described within the ongoing challenge of “missing ice” in ref. ^62^ For our CESM1 simulations, the paleotopography and ice sheets represent the time periods 13,000 BP for BA and 12,000 BP for YD as displayed in Fig. 1.

### Boundary conditions and model setup

To setup CESM1 for simulations of the last deglaciation, we adjusted all horizontal boundary files of the different modules accordingly. This means that standard pre-industrial boundary condition files (F1850 case) from CESM1.0.5 were interpolated onto the new paleotopography provided along with GLAC-1B as described in ref. ^63^ The surface types of new land areas that were added due to the low sea-level stands of the BA and YD periods were created by next-neighbour interpolation with the exception of glaciated areas in GLAC-1B, which were set as glacier-based land units on the ice mask from GLAC-1B. This means that similar to other state-of-the-art model simulations of the deep past (e.g. ref. ^53^), vegetation is kept constant to pre-industrial conditions and urban areas are set off.

The paleo-ocean state was prescribed in our simulation using monthly mean surface temperatures and the sea-ice fraction for BA and YD from a previous transient atmosphere–ocean simulation of the last 21 kyr with CCSM3^25,26,33^. The global fields for the monthly mean sea-surface temperatures (SST) and ice fraction climatology for BA and YD were calculated based on periods of 100 years centred around the model years 13 kyr BP (BA) and 12.17 kyr (YD), respectively. Based on the CCSM3 simulation, the YD ocean state reflects a weakening of the AMOC of ~5.27 Sv (−36.3%) from ~14.5 Sv during BA to 9.2 Sv during YD reflecting the hosing experiment with the best available agreement with paleoclimate data so far^25,26^.

### Experimental design and radiative forcing

Using the paleotopography and surface ocean conditions for BA and YD, we run CESM1 for 150 model years per time-slice and use the last 100 model years for analysis. As the initial conditions are unknown, the model was initialised from default conditions (1987-01-01) for CAM while a cold start was used for CLM allowing the model to adapt to the new topography and the presence of continental ice sheets. While the atmosphere adapts quickly to the new conditions within around 10 years, deep soil temperatures at 35 m depth are reasonably stationary only after around 50 years and represent the slowest variable of the coupled atmosphere–land-ice-model configuration to reach a new equilibrium.

Consistent with the prescribed surface ocean climatology calculated from CCSM3, radiative forcing is set to 13 and 12.17 kyr BP, respectively. The orbital changes during the late deglaciation are characterised by high and rapidly increasing summer insolation over high northern latitudes. As a consequence, a notable additional positive radiative forcing exists for the mid-YD during northern summers with +5 W/m^2^ (June, 60°N) as compared to BA.

GHG were calculated as an average of ±50 years around the orbital years using merged values derived from air bubbles trapped in Greenland and Antarctica ice cores^64^. The GHG data is hence identical to the values used in the CCSM3 simulation^25,26^. The radiative forcing ΔF (W/m^2^) derived from changes in GHG are rather small for YD relative to BA. Using empirical equations derived from atmospheric radiative transfer models (Table 6.2 in ref. ^65^), the GHG concentrations can be transferred to Δ*F*. A total Δ*F* of −0.907 W/m^2^ for BA and −1.086 W/m^2^ relative to the reference values of 1750 AD leads to a small negative radiative forcing (Δ*F* = −0.180 W/m^2^) during the YD relative to BA in terms of GHG. Using compound-specific derivations of Δ*F* for CO_2_, CH_4_ and N_2_O ^66^, the negative radiative forcing represents a total reduction in terms of CO_2_ equivalent of only −7.75 ppm. GHG concentrations and the derived compound-specific changes in radiative forcing are listed in Supplementary Table 1.

### Proxy data

We compiled and evaluated published European chironomid, coleoptera, aquatic pollen and plant macrofossils records, which are common proxies for summer temperature. As several of the insect data sets have already been used in previous studies, including YD model-data comparisons (e.g. refs. ^10,11^), the methodology to derive quantitative temperature reconstructions is only briefly discussed below. A different and independent approach to reconstruct summer temperatures, which is used in this study, relies on aquatic and terrestrial climate indicator-plant species (micro- and macrofossils) extracted from lake sediments^41^. The various data sets and the methodological approach used here to derive summer temperatures are explained in more detail.

### Insect-based July temperature reconstructions

Quantitative temperature reconstructions based on fossil chironomid and coleoptera assemblages preserved in lake sediments (Supplementary Data 1) reflect July or more generally summer mean (May–August) temperatures, respectively. Chironomid-inferred reconstructions are based on mathematical transfer functions that convert the fossil biological information into a specific climate parameter^67^. By contrast, reconstructions based on coleoptera rely on the mutual climatic range (MCR) method^68^, which has been extensively applied to beetle assemblages in Europe^69^. This technique leans upon the assumption that today’s climatic tolerance range of a species can be applied to fossil beetles, whereby a tolerance range can be assigned to the occurrence of a given species in geological records. The proxy-based temperature reconstructions included in this study are reported according to the original publications (Supplementary Data 1). For details on the specific transfer function or the MCR method used to transform proxy data into quantitative estimates, the reader is referred to the original studies (Supplementary Data 1). The temperature values of the assembled chironomid and coleoptera data sets are according to those published by the respective authors.

To define the boundaries for BA and YD periods in the stratigraphic records, we generally adopted the pollen stratigraphic zonation presented in the original publication. Where no pollen stratigraphic diagrams were available, we relied on other biostratigraphic information (e.g. changes in plant macrofossil, chironomid or diatoms assemblages). In the absence of biostratigraphic indicators, we used the available ^14^C-chronologies to define the climatic boundaries in comparison with correlative events in NGRIP ice cores (e.g. GI-1abc and GS-1)^70^. Independently from the used definition for the boundaries, they are clearly visible in the time series and/or stratigraphy.

We deem relying on the local pollenstratigraphy as the most conservative and suitable way to define the transition into the cold YD stadial. Indeed, this provides time slices that can be consistently compared to model output from our simulations, since the definition of the BA-YD transition in the model was defined by the start of SST cooling in the Nordic Seas.

It should be noted however that the time-slice comparison averages out and hence ignores climatic variations within the periods of BA and YD, respectively. Some studies suggest a partial resumption of the AMOC during some parts the YD leading to a stepwise or at least bi-partite structure of the YD^15,71,72^ with a more stable early YD and more unstable late YD^71^ with a mid-YD recovery^71,73,74^. As we focus on the dominant changes in the mean states between BA and YD, the time evolution and variability around the mean states are not considered here but should be kept in mind when studying transient changes or the stability of the AMOC, e.g. during the YD.

### Climate indicator-plant species approach

Here we compiled all available plant indicator species (plant macro remains, local pollen) from published sources in Europe. In limited cases, when no aquatic or other indicative riparian, emergent, or helophyte data were available, macro remains of tree species were used to infer quantitative summer temperature. These are indicated in Supplementary Data 1.

We use the approach developed by Väliranta et al.^41^, where current plant species distribution data are linked to measured meteorological data in Finland to quantitatively reconstruct July temperatures. Finland is relatively flat with low mountains only in the northwest. The oceanity–continentality gradient is not particularly marked today: in general, continentality increases from the southwest (semi-oceanic) to the northeast (semi-continental). Likewise, the precipitation gradient is gradual, with a difference of ca. 200 mm/a between southern and northern Finland. The latitudinal range is over 10°, resulting in a pronounced south–north temperature gradient. The *T*_jul_ ranges from ca. 17 °C in the south–southeast to ca. 7.5 °C in the mountains of western Lapland. As a result, Finland spans several bioclimatic zones from the boreo-nemoral to the boreal and to the subarctic vegetation belts. Therefore, many plant species reach their northern distribution limits within Finland. Only in the northernmost part of the country are plant distributions limited by elevation-related climatic conditions (orohemiarctic). Unlike many European countries where human activities strongly influence plant distributions, Finland can be considered to be in a relatively natural state. These geographical facts provide an excellent setting to exploit observed modern species–temperature relationships for palaeoecological temperature reconstructions. A unique modern species-specific spatial plant distribution data set (http://www.luomus.fi/kasviatlas) covers the whole of Finland and long-term meteorological climate normals are readily available. These data come from 10×10 km grid cells. Thus, the plant distribution database, based on continuous botanical surveys, can be used to correlate modern species distributions with climate variables.

It can be argued that the modern species–temperature relationship may be affected by non-stationarity, which has for example been discussed for the calibration of hydroclimate/temperature proxies during the recent past, such as trees (e.g. refs. ^75,76^). Although this cannot be ruled out, the relatively simple approach to link the presence of a plant species to its minimum July temperature requirement is fairly robust also in the past for temperature sensitive plant species once the population had become established during the BA. A major advantage of using aquatic or shoreline plants as done in our study is the constant availability of a water source. Compared to remote terrestrial proxies such as tree pollen, local plants that grew close to the lake or at the lake shore are much less likely affected by changes to drier conditions and hence less prone to non-stationarity which likely affects hydroclimate proxies^76^; e.g. due to direct or temperature-induced drought stress, changes in snow melt, low soil humidity or changes in seasonality^75^.

A recent study for the mid-Holocene suggests that tree pollen in the Mediterranean yield a cold bias for summer temperatures in comparison to chironomid-derived temperatures owing to drier conditions^77^. This would imply that tree pollen for the drier climate of the YD with a higher summer radiation are cold biased too. This may explain some of the discrepancy relative to the considerably warmer YD plant indicator species derived temperatures in our study.

To reconstruct July temperatures based on reported species assemblages from fossil records, we chose the species that currently has the highest July temperature requirement and combined this information with the measured modern (observations 1970–2000) mean July temperature values at the modern northern species distribution limits in Finland. For each species, we used interpolated mean July temperatures over a 10×10 km grid cell and several grid cells containing species occurrences along the current distribution boundary were analysed. A median and a July mean range, i.e. the lowest and the highest value along the species-specific northernmost distribution boundary of these grid cells is given in Supplementary Table 2. The median value integrates all July temperature values, i.e. also for those occurrences, which may be situated in unusually favourable microhabitats due to exceptionally ideal microclimates. It should be kept in mind that modern plant distribution maps typically show a pattern where the plant-specific minimum July requirement increases towards more continental areas, i.e. the distribution area shifts southwards. Conversely, in oceanic areas species distributions run northwards. This pattern may lead to underestimated July reconstructions for continental areas and overestimations for highly oceanic areas.

Like macroscopic plant remains, pollen of aquatic and shoreline species are only locally dispersed^78,79^ and thus represent local rather than regional conditions. However, pollen of aquatic and riparian species are not always reported or published, and these species are always excluded from the temperature reconstructions. Aquatic and riparian pollen taxa are often scarcely present and thus, similarly to macrofossil records, their records are often not continuous. Therefore, the temperature reconstruction is based on presence only (not on abundance) of these species. Moreover, the absence of macroscopic plant remains or local pollen from fossil subsamples is not a solid proof of absence of the plant itself, while the presence of macroscopic species remains or local pollen can be taken as a strong evidence of actual presence in situ.

The discontinuous nature of the data and the indicator species approach, only accounting presence, prevent the estimation of a sample-specific error provided for instance by transfer function procedures. Moreover, it should be noted that our reconstruction is based on northernmost individual occurrences, while the main populations are always further to the south. The derived temperature values are therefore minimum July temperatures and may indicate colder than the actual July mean temperatures. The order of possible July temperature overestimations can be calculated from the differences between the “median observed” and the “lowest observed” July temperature (Supplementary Table 2). These median-to-lowest differences vary from 0.1 to 3.3 K for different plant species. Based on the 35 plant species in Supplementary Table 2, for which such a plant-specific uncertainty can be derived, the mean plant-specific uncertainty for common vs. coldest minimum July temperature (median minus minimum) is around 1 K.

A total of 38 taxa, which have value as temperature indicators, were compiled from the literature. These species and their current geographical distribution with corresponding July temperature in Finland are presented in Supplementary Table 2. As the chronological framework for most of these data sets was poor and mainly based on pollen stratigraphic assignments, we grouped the available taxa and assemblages at each site into two time periods, BA and YD. This approach prevents the identification of site-specific temporal variability, but is the only possibility given the lack of chronological constraints. It is also well suited for the time-slice comparison of steady state simulations of BA and YD in this study.

### Taxonomic notes and potential sources of error

The species *Elatine hexandra* does not occur in Finland. Its northernmost distribution is in southern Sweden and probably corresponds to a July temperature of >16 °C. As there is no comparable meteorological/species distribution data available for southern Sweden, we assigned a cautious July temperature estimate of 15.7 °C, i.e. the same as for *Typha*.

In Finland *Najas marina* and *Potamogeton pusillus* live in brackish water environments. On the European mainland, these species have an inland and temperate zone distribution. We nevertheless used their distribution in Finland to infer July temperatures. *Zannichellia palustris* has a complicated distribution. In Fennoscandia it thrives in brackish water and individual occurrences are found quite far north. However, its main distribution area is in the European inland temperate zone and in freshwater environments. Also for this taxa, we used its distribution in Finland to infer July temperatures.

The taxonomy for *Potamogeton* has changed through time. For example, the species which was earlier called *P. pusillus*, is currently named as *P. berchtoldii* with a minimum July requirement of 12.2 °C. In turn, the northernmost distribution range of the species which is nowadays called *Potamogeton pusillus*, corresponds to a July temperature of 13.6 °C. In cases where the taxonomy of *P. pusillus* was uncertain, we used the lower value of 12.2 °C.

If the used pollen reference indicated *Typha angustifolia* type, we used a temperature reconstruction of 10 °C, because the pollen taxon *Typha angustifolia-*type includes several *Sparganium* species. Sometimes this pollen taxon was indicated as *Typha Sparaganium*. If the pollen reference reported *T. angustifolia* or *T. latifolia*, the temperature requirement was set to 15.7 °C.

### Proxy data compilation

To compare the quantitative multi-proxy temperature reconstructions with our time-slice simulations for BA and YD, we consider all published plant and insect paleo-data sets covering the BA and/or YD across Europe (Supplementary Fig. 1). The paleo-proxy compilation contains a total of *N* = 122 records which were subdivided into two proxy groups.

The first group with in total *N* = 34 records (Supplementary Data 1) consists of insects with chironomids (*N* = 28), cladocera (*N* = 1) and coleoptera (*N* = 5). These records are mostly characterised by high-temporal sampling resolution and provide relatively continuous time series. However since we are here only interested in the general mean temperature difference between the BA and YD, we ignore within-period temperature variations or differences at the onset and termination of YD and BA. We therefore calculated averages for BA and YD using the biostratigraphic and/or chronozones provided in the original publications as boundaries. Three out of the 34 records only covered one of the two investigated time periods and could thus not be used for comparison of temperature anomalies with our time-slice experiments. They are however used for the evaluation and comparison of mean temperatures between both proxy groups for BA and YD.

The second group, comprising plant indicator species, provides only discontinuous time series with low temporal resolution. Rather than using averages, temperature estimates in group 2 are based on the presence of the species with the highest temperature requirements. This second group consists of *N* = 88 records covering the BA and/or YD (Supplementary Fig. 1) and is separated into aquatic (P, a) or emergent pollen (P, e), plant macrofossils from aquatic (M, a) or emergent (M, e) species, and tree macrofossil remains as only terrestrial indicator (M, t) (see Supplementary Data 1). Several lake records do not cover both periods, which reduces the total number of available data points for the comparison of temperature anomalies to *N* = 42. All available records are however used for the evaluation and comparison for the mean temperature ranges for BA and YD as shown in Supplementary Fig. 1.

Together, the *N* = 31 records from group 1 and *N* = 42 records from group 2, provide us with an *N* = 73 paleo-temperature data set that allows contrasting the climate states of BA and YD. For the multi-proxy temperature compilation of the mean temperature values and differences for BA and YD, the reader is referred to Supplementary Fig. 1 and the Supplementary Data 1.

### Uncertainties of July temperature proxy compilation

The proxy-based July temperature compilation presented here for the YD and BA is subject to two main sources of uncertainty: the reconstruction of July temperatures itself and the time attribution of samples to belong to the respective periods of YD or BA.

In case of group 1 (chironomids, coleoptera, cladocera), the compilation of records is entirely based on previously published July temperatures. On the basis of these studies (references in Supplementary Data 1), typical uncertainty estimates for European chironomid-based temperature reconstructions yield a standard error of prediction of ~1.3 K with a range of 1 K to 1.6 K (see site-specific references in Supplementary Data 1). On the basis of a recent synthesis project for European chironomid records^10,11^, the statistical uncertainty for YD-BA July temperature anomalies has been estimated following a strict protocol. On the basis of this approach, sample-specific errors were estimated to be around 1.4 to 1.5 K with an overall RMSE of prediction of 1.4 K for chironomid-based July temperature anomalies. Although the uncertainty of 1.4 K reflects only the uncertainty of the used transfer functions relative to a modern calibration data set, it has been assumed to be representative for the uncertainty of YD-BA anomalies^11^.

It is a common problem for basically all empirical ‘modern analogue’ approaches that it is unclear to which extent the established modern species–temperature relationship is maintained under fundamentally different conditions like e.g. the deglaciation or stadial conditions like the YD. In case of chironomids, multiple studies have shown that other factors than July temperatures incl. changes in seasonality can have a comparably high correlation with chironomid assemblages^37–40^. Based on our climate simulation, the extreme change in seasonality with a strong cooling lasting until May or June (Fig. 4c) may partly override the modern-day dependency of chironomid assemblages on July temperatures.

In case of July temperatures reconstructed from climate indicator-plant species (group 2), the method and hence the error estimates and interpretation of the reconstruction is exactly the same as in the previous reconstruction of early Holocene temperatures^41^. Although the indicator species approach does not provide sample-specific error estimates and hence a definition of statistical uncertainty for the July temperature reconstruction, there is a solid understanding of estimating sources of uncertainties and the risk of bias.

As shown in Supplementary Table 2, the largest conceivable plant-specific uncertainty of minimum July temperature estimates, following the definition of the median-to-lowest difference^41^, ranges from 0.1 to 3.3 K. The mean uncertainty based on the 35 taxa in Supplementary Table 2 is ~1 K (median 0.7 K). Because the northern distribution limit of a plant species reflects only individual species while most plants grow further south at warmer temperatures, the minimum mean July temperature is rather a conservative (lower) estimate than the mean July temperature. In addition, because the indicator species approach based on plant macrofossils relies on rare presence of the warmest found plant species, there is a clear risk to underestimate July temperatures in the reconstruction. Although the presence of a plant in situ is a strong proof that it was at least as warm as the related plant-specific July (*T*_July_ ≥ *x*) temperature (true presence), it cannot be ruled out that it was even warmer but no such plant indicator species was found (the problem of accounting for false absence). Both, the conservative definition of plant-specific July temperatures and the biased risk of false absence of warmer species in the sediment record, are a clear indication that the indicator species approach used here represents rather too low (potentially cold biased) July temperature estimates. Although this uncertainty cannot be quantified statistically, this implies that the difference between chironomid-based vs. plant-based July temperatures might be even larger than estimated by this study.

Another source of uncertainty is related to the question whether the samples used to reconstruct July temperatures of a given time period BA or YD might actually contain samples coming from another time period instead. For our compilation we rely here entirely on the definition of boundaries as provided by the original publications (see references in Supplementary Data 1). Although no statistical age uncertainty can be estimated from these studies, it should be noted that chironomid records differ qualitatively from plant macrofossil records in terms of age representation.

In case of chironomids, the uncertainty that the mean July temperature of a time sample YD or BA is compromised by a false time attribution of some samples at the boundaries is rather small. Most time series are relatively continues with a relatively high sampling resolution (often at least ~100 years) and show a clear abrupt cooling signal at the beginning and end of the YD (see references in Supplementary Data 1).

In contrast to chironomid records, the reliance on rare presence, and hence the discontinuous nature of climate indicator-plant species records, makes the time attribution of samples more sensitive to potential errors at the boundaries. While we rely here on the definition of boundaries presented by the original publications, potential errors cannot be ruled out. The best way to minimise the risk of false time attribution at the boundaries is to rely on a large data set of records from different locations. Based on the compilation presented here, 25 out of 42 records (~60% of sites) show no change in July temperatures for YD-BA. As a result, the median of 0 K temperature change for YD-BA anomalies is fairly robust to potential errors which may be introduced by a subset of locations with doubtful samples at the boundaries.

### Data availability

The compilation of multi-proxy data used in this study are provided as Supplementary Data 1. Climate model data are available from the corresponding author upon reasonable request.

## Electronic supplementary material

Supplementary Information

Description of Additional Supplementary Files

Supplementary Data 1
