# Supplementary material for: Warm summers during the Younger Dryas cold reversal
Source: Nat Commun. 2018 Apr 24;9:1634. doi: 10.1038/s41467-018-04071-5 (PMC5915408; doi:10.1038/s41467-018-04071-5)
Supplement: Supplementary file 1 — Supplementary Information [file 41467_2018_4071_MOESM1_ESM.pdf]

Supplementary Information

Warm summers during the Younger Dryas cold reversal

Schenk et al.

Supplementary Table 1: Greenhouse gas (GHG) concentrations for BA and YD. The GHG concentrations and the resulting changes in radiative forcing ΔF for YD-BA used in our CESM1 simulation are consistent with the previous transient simulation of the same time periods performed with CCSM3 [25, 64]. For the calculation of ΔF, see Methods.

**GHG forcing**	**BA (13 kyr)**	**YD (12.17 kyr)**	**ΔF of YD-BA**
**CO_2**	237.8 ppm	238.4 ppm	+0.013 W/m^2
**CH_4**	633.6 ppb	475.9 ppb	-0.107 W/m^2
**N_2O**	265.2 ppb	241.3 ppb	-0.085 W/m^2
**Total ΔF YD-BA**	-7.749 ppm CO_2-equivalent		-0.180 W/m^2

Supplementary Table 2: List of late-glacial plant species and related July temperatures. The species include plant macrofossil and aquatic pollen data with related minimum July temperature ranges determining the species' current northernmost distribution limit [in °C] as defined by ref. 41 (Methods).

Taxon	Median of July mean	July mean range	Taxon	Median of July mean	July mean range
Alisma plantago-aquatica	14.3	14.1-14.9	*Myriophyllum spicatum group*	12.2	10.7-13.3
Calla palustris	13.6	13.3-14.0	*Myriophyllum verticillatum*	13.8	13.5-14.2
Callitriche cophocarpa	13.7	13.5-13. 9	*Najas flexilis**	16.8*	16.7-16.8
Callitriche hermaphroditica	14	13-14.5	*Najas tenuissima*	16.6	16.1-16.7
Callitriche spp.	11.9	10.0-12.5	*Nuphar*	13.1	12.5-14.0
Campanula latifolia	15.7	15.0-16.0	*Nymphaea*	13.5	13.2-14.0
Carex pseudocyperus	16.7	15.7-16.8	*Potamogeton berchtoldii*	12.2	10.0-13.6
Ceratophyllum	14.1	13.6-14.3	*Potamogeton crispus*	16.5	16.2-16.8
Cicuta virosa	13	12.1-14.1	*Potamogeton filiformis*	13.5	10.5-15.4
Cladium mariscus	16.0*	15.7-16.3	*Potamogeton lucens*	14.2	13.9-14.5
Elatine hydropiper	14.2	13-15	*Potamogeton natans*	12.9	12.2-13.8
Fragaria vesca	13.3	12.0-14.2	*Potamogeton obtusifolius*	13.9	13.6-14.3
Glyceria notata	16.7*	16.5316.8	*Potamogeton praelongus*	13.2	10.0-13.9
Isoëtes lacustris	12.7	10.9-13.0	*Potamogeton pusillus****	13.6	
Isoëtes echinospora	12.4	9.1-13.0	*Potaogeton spp.*	11.7	10.1-13.6
Lemna minor	13.6	12.3-14.3	*Subularia aquatica*	11.4	10.1-12.7
Littorella uniflora	16.1	14.3-16.8	*Trifolium repens*	12.5	10.4-12.9
Lythrum salicaria	14.3	14.2-14.9	*Typha*	15.7	15.1-16
*Menyanthes trifoliata***	8 (10)		*Zannichellia palustris****	14.4	

*Average value based on two occurrences only.

** Temperature value based on literature [79]. However, 10°C is a more realistic July value because the species does not grow beyond the tree line in Finland.

*** The species has a complicated current distribution pattern. In Finland the species mainly lives in brackish water environments. Only scarce occurrences elsewhere. In European mainland the species has an inland and temperate zone distribution. Thus, the temperature estimate remains tentative.

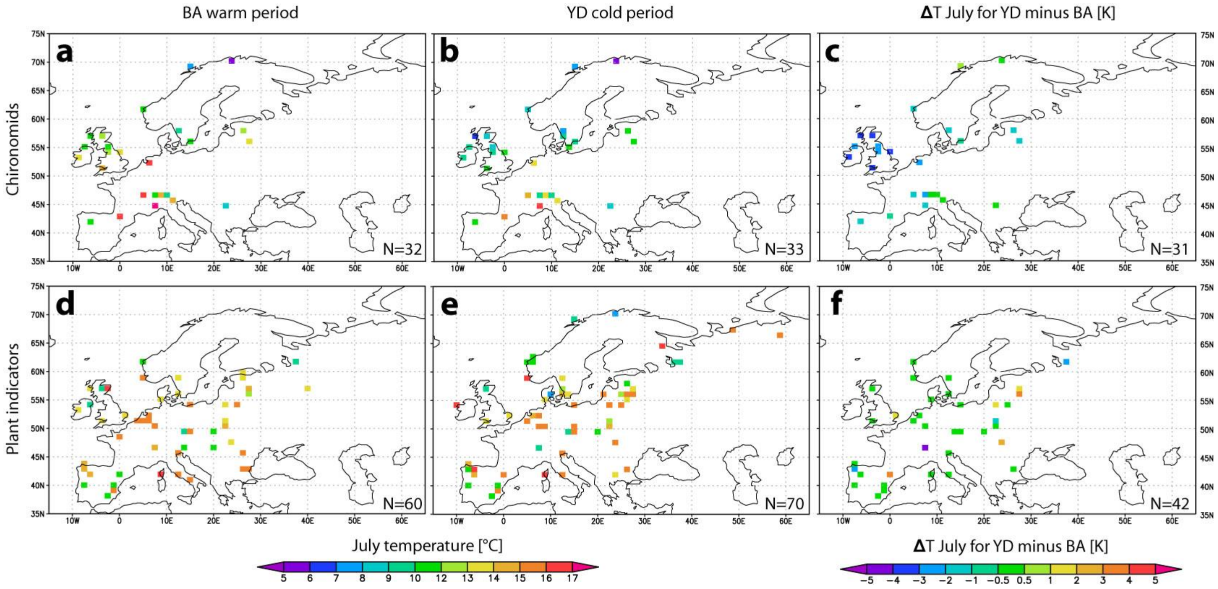

Supplementary Figure 1. Spatial distribution of proxy-based mean July temperature reconstructions. (a) mean July temperatures [°C] for BA and (b) the YD period and (c) the respective mean difference of YD-BA [K] for group 1 (Chironomids, Cladocera and Coleoptera). (d), (e) and (f) for group 2 (plant indicator species). The number N of available sites is indicated in the lower right corner of the maps. For more details about site descriptions and original sources, see Supplementary Data 1.

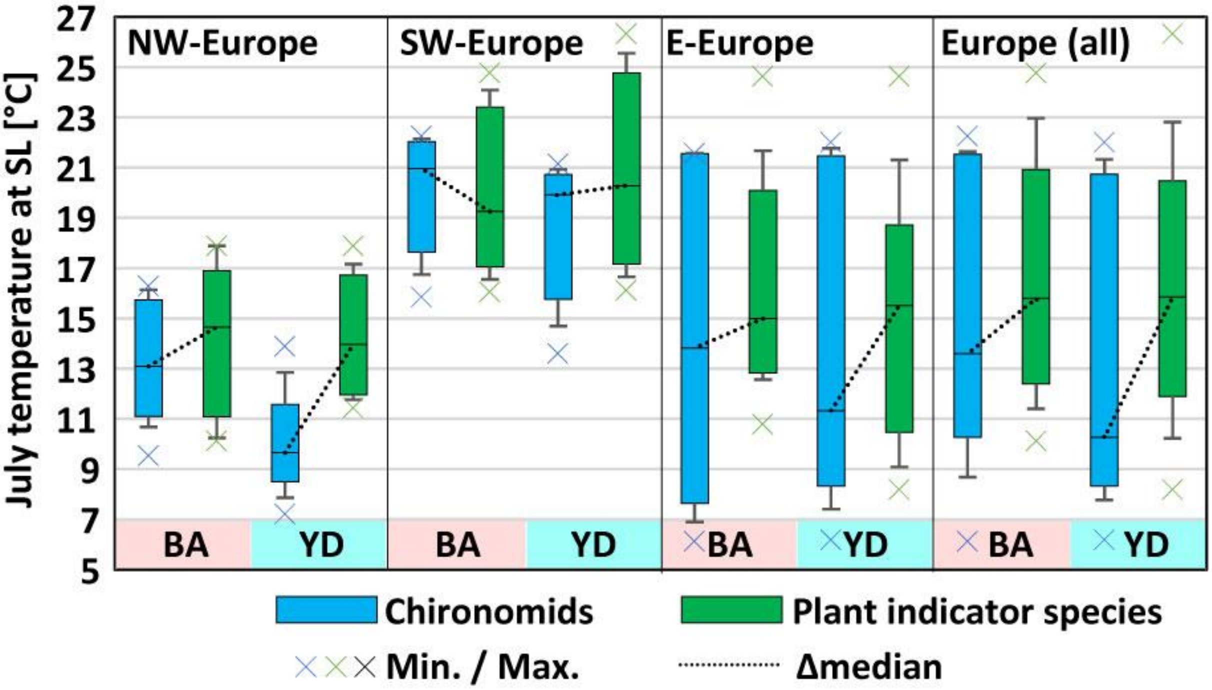

Supplementary Figure 2. Comparison of regional elevation-corrected mean July temperatures derived from both proxy types for BA and YD. Boxes represent 80% of the spatial range of regional temperatures, whiskers the 90% spread and X the min./max. values. July temperatures are corrected to sea-level (SL) for comparison assuming a lapse rate of 0.65K per 100 m. Site elevations are given in the Supplementary Data 1. Elevation-corrected median July temperatures are systematically warmer for plant indicator species than for chironomids with exception of SW-Europe during BA. A direct comparison of the regional temperature range is not possible due to regionally different numbers and spatial distributions of lake sites.

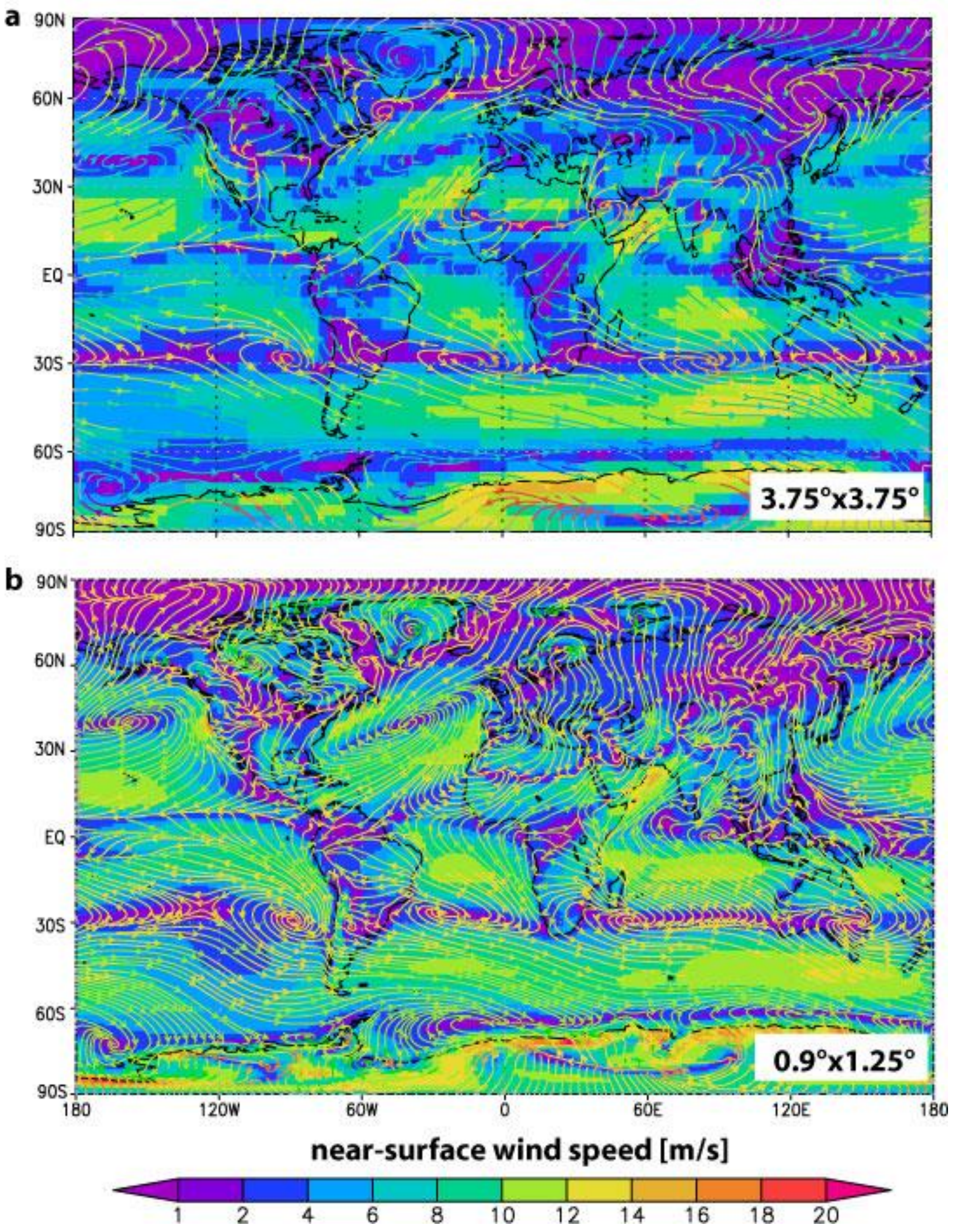

Supplementary Figure 3. Comparison of monthly mean near-surface wind speeds [m/s] for July during the YD simulated by (a) CCSM3 (3.75°x3.75°) and (b) CESM1 (0.9°x1.25°). Notable differences in atmospheric flow (stream lines) are evident over Europe in response to continental ice sheets. CCSM3 simulates westerly flow across Europe while meridional flow is simulated at high resolution by CESM1.

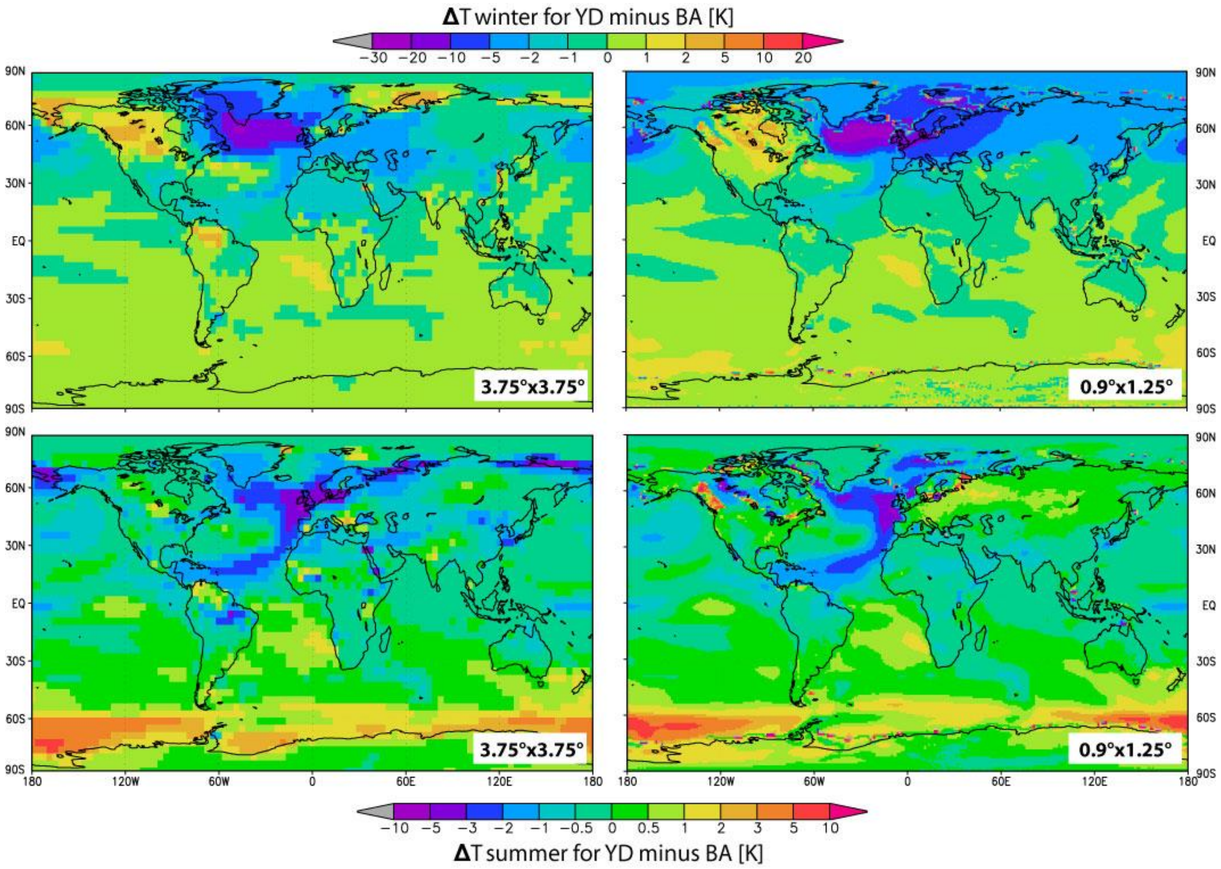

Supplementary Figure 4. Comparison of simulated ΔT of YD minus BA for CCSM3 and CESM1. Seasonal mean temperature difference for winter (DJF, top) and summer (JJA, bottom) as simulated by TraCE [25] (left) and CESM1 (right) for 100 model years straddling 13.000 and 12.170 BP, respectively. Note that the SST and sea-ice fraction is identical in both simulations while temperatures over sea-ice and land differ for CESM1. Different scaling is used for ΔT winter and ΔT summer.

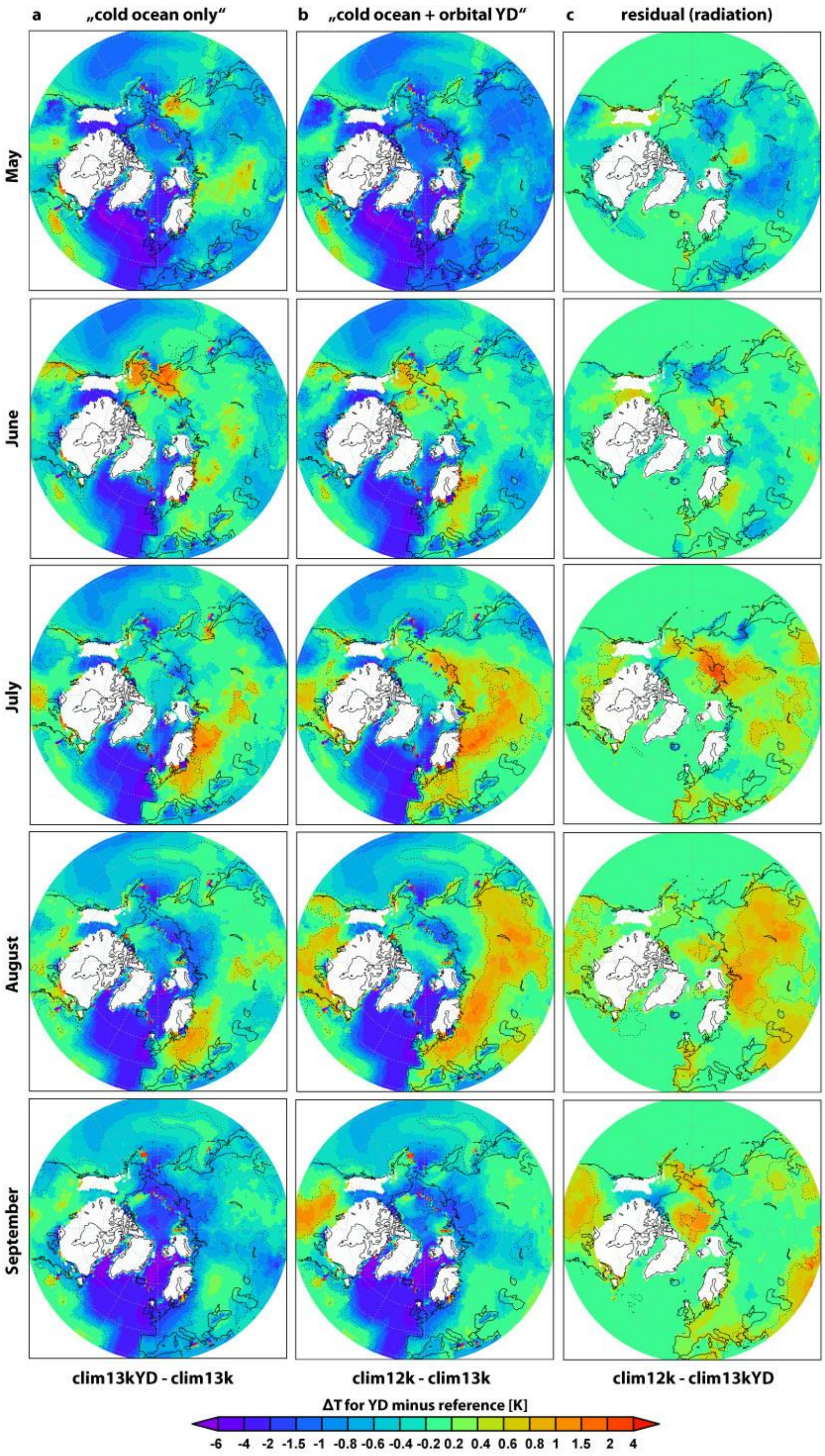

Supplementary Figure 5: Comparison of simulated ΔT relative to the warm state of BA (clim13k) for two Younger Dryas experiments. (a) “cold ocean only” sensitivity experiment with mid-YD ocean state but unchanged radiative forcing of 13 ka (clim13kYD) and (b) mid-YD with realistic radiative forcing (clim12k). (c) shows ΔT between a mid-YD ocean state with and without radiative changes (residual).
