# Supplementary material for: Warm summers during the Younger Dryas cold reversal
Source: Nat Commun. 2018 Apr 24;9:1634. doi: 10.1038/s41467-018-04071-5 (PMC5915408; doi:10.1038/s41467-018-04071-5)
Supplement: Supplementary file 2 — Description of Additional Supplementary Files [file 41467_2018_4071_MOESM2_ESM.pdf]

Description of Additional Supplementary Files

File Name: Supplementary Data 1
Description: Data compilation of multi-proxy July temperature reconstructions.
